# Nonhost Disease Resistance in Pea: Chitosan’s Suggested Role in DNA Minor Groove Actions Relative to Phytoalexin-Eliciting Anti-Cancer Compounds

**DOI:** 10.3390/molecules25245913

**Published:** 2020-12-14

**Authors:** Lee A. Hadwiger

**Affiliations:** Department of Plant Pathology, Washington State University, Pullman, WA 99164-6430, USA; chitosan@wsu.edu; Tel.: +1-509-335-3751; Fax: +1-509-335-9581

**Keywords:** chitosan, DNA minor groove, chromatin, nonhost disease resistance, pea, *Fusarium solani*, defense genes

## Abstract

A stable intense resistance called “nonhost resistance” generates a complete multiple-gene resistance against plant pathogenic species that are not pathogens of pea such as the bean pathogen, *Fusarium solani f. sp. phaseoli* (Fsph). Chitosan is a natural nonhost resistance response gene activator of defense responses in peas. Chitosan may share with cancer-treatment compounds, netropsin and some anti-cancer drugs, a DNA minor groove target in plant host tissue. The chitosan heptamer and netropsin have the appropriate size and charge to reside in the DNA minor groove. The localization of a percentage of administered radio-labeled chitosan in the nucleus of plant tissue in vivo indicates its potential to transport to site(s) within the nuclear chromatin (1,2). Other minor groove-localizing compounds administered to pea tissue activate the same secondary plant pathway that terminates in the production of the anti-fungal isoflavonoid, pisatin an indicator of the generated resistance response. Some DNA minor groove compounds also induce defense genes designated as “pathogenesis-related” (PR) genes. Hypothetically, DNA targeting components alter host DNA in a manner enabling the transcription of defense genes previously silenced or minimally expressed. Defense-response-elicitors can directly (a) target host DNA at the site of transcription or (b) act by a series of cascading events beginning at the cell membrane and indirectly influence transcription. A single defense response, pisatin induction, induced by chitosan and compounds with known DNA minor groove attachment potential was followed herein. A hypothesis is formulated suggesting that this DNA target may be accountable for a portion of the defense response generated in nonhost resistance.

## 1. Introduction

The current case presented for chitosan being a DNA minor groove-signaling elicitor is based on information from both previous [1,2] and newer reports [3] of how comparatively short AT/GC sequences recognize DNA-specific compounds (e.g., netropsin). The data suggest parallel hypotheses of defense gene-inducing mechanisms occurring in vivo within pathogen-challenged plant tissue [4].

DNA minor groove-specific compounds mimic normal elicitor components such as chitosan oligomers (heptamer) that move between a fungal pathogen and plant host tissue inducing disease resistance responses [5]. The production of pisatin, a pea phytoalexin, is the major disease resistance response monitored herein. Features of the induced chromatin alterations include the binding of the positive elicitor charge with the negative charges of DNA, recognition of DNA sequences, and the hypothesized expansion of the minor groove occurring in the bound DNA. These changes are likely part of the more dramatic actions of other pisatin inducers that cleave DNA strands or intercalate between DNA base pairs [4,6] that can activate the “nonhost resistance” defense response of plants [5,7]. The current case study is an assimilation of long-term research relating how fungal signals develop nonhost resistance in pea tissue, monitored here as pisatin production. Pea endocarp tissue is not a host to the bean pathogen, *Fusarium solani f. sp. phaseoli* (Fsph), and actively resists it. Chitosan originating from the fungal cell/cell wall is a major natural compound signaling the pea’s defense response [5,7]. The chitosan as a phytoalexin-inducing signal causes DNA conformational changes to chromatin due to the proposed targeting of the DNA minor groove [4] and in vitro complexing alters DNA CD and UV monitored melting spectra [2].

Chitosan is not the sole elicitor of pea endocarp phytoalexin production in nature [7,8] and pisatin is not the sole component of the growth-suppressing action of the nonhost resistance response. Suppressive action is also related to salicylic acid (SA) [8], DNase [9], reactive oxygen species (ROS) [10], defensins [11,12], and other physiological responses [7,13] in plant defense. The ability of the plant tissue to respond to an array of fungi, bacteria and other living cells indicates the existence of a large source of defense elicitors, and correspondingly, there is both a diversity of signal receptors and probably multiple paths toward the cellular sites of transcription within the host [7,13,14].

Chitosan as a polymer of glucosamine residues is highly positively charged and thus can rapidly attach to many negative components within plant cells. Past research has examined potential signal actions of released fungal pathogen elicitors on cell surface/cell membrane receptors [14]. The strong positive charge of chitosan or its modifications formulates its affinity to many negative proteins, thus confounding the identification of a single signal receptor [15]. Plant proteins have been reported as plant receptors of chitosan [14]. The necessary “in turn” actions of signaling cascades directed toward the site of transcription are mostly unresolved. In contrast, the positive charges of chitosan enable it to attach directly to DNA. Its route to the plant nucleus has been monitored with radio-labeling and anti-chitosan antisera [1]. The chitosan heptamer after traversing the cell membrane has been hypothesized to reside in vitro in the DNA minor groove [4]. This potential in vivo DNA minor groove association is shared by pharmaceutical compounds such as netropsin and distamycin A. The specifics of in vitro associations of netropsin and distamycin with DNA, DNA base sequences, and minor groove insertion have been examined in detail [16,17,18,19,20,21].

**Figure 1:**
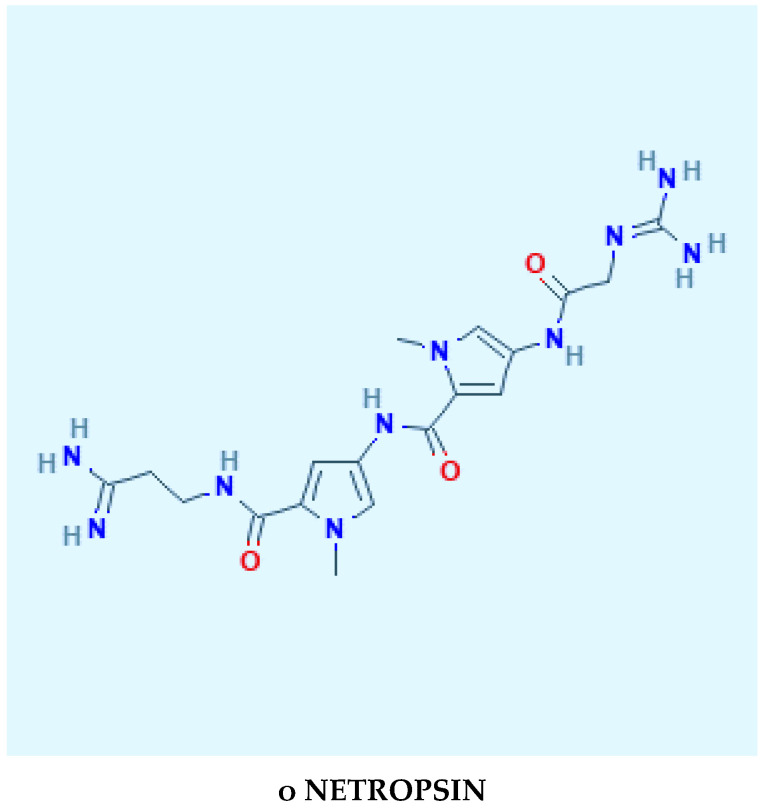

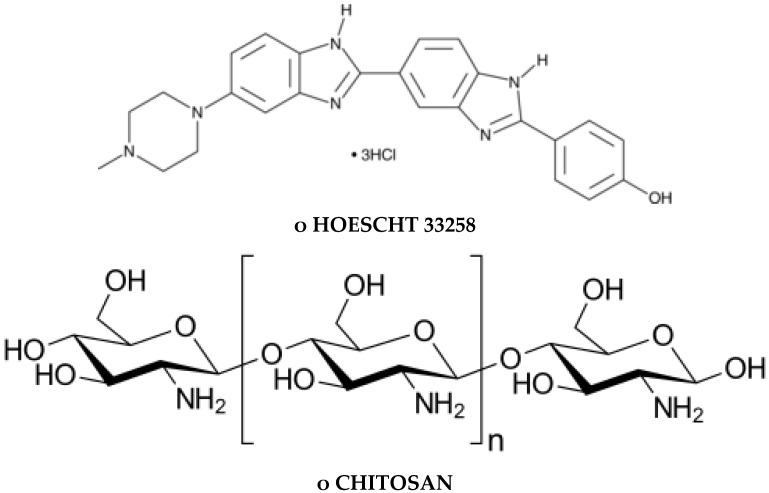
Structural formulas of compounds with a potential to complex with the DNA minor groove. Structure formula origin from PubChem-NIH.

An example of compounds that complex with specific sequences of DNA within the minor groove is shown in Figure 1 [17].

DNA minor groove-binding compounds have been developed primarily in search for anti-cancer drugs. The compounds can also be anti-microbial and anti-viral with properties that result in the inhibition or activation of transcription in targeted cells [19]. Minor groove binders have features such as curved shapes and groups with positive charges [20]. Additionally, they have an H-bond donating ability for flat conformation with the flexibility to adjust to the minor groove [21]. DNA AT-rich sequences are often preferred such as AATT. Based on this preference the following is an example of the presence of such receptor AATT sites [19] in the region preceding the start site of the pea HMG A gene coding for the high mobility protein that acts as an “architectural” transcription factor [22].

### 1.1. AT-Rich DNA Sequences in Pre-Promoter Region of Pea HMG A Gene

TCAATTAAAA AATCAATTTA TTTATTTCAT TTCATAAATA TATTCATAA
AATTAAATAC AATGAGTAGA ATTTCAAAC TCTCAATAAATTTTAGT

### 1.2. Sequences Used in Crystal Modeling of Drug/DNA Minor Groove Complexes

Netropsin: d(CGCAATTCGCG)
Distamycin: d(CGCAAATTTGCG)
Hoechst 33258: d(CGCGAATTCGCG)

### 1.3. DNA Minor Groove Compounds Evaluated as Pisatin Elicitors Include Chitosan, Chromomycin A3, Distamycin, Hoecsht 33258, Netropsin, and Methyl Blue

Most of the DNA minor groove attaching molecules (except chromomycin) above possess positively charged groups that assist in complexing AT-rich regions of pea DNA. AT-rich regions are abundant within plant DNAs making many potential DNA minor groove attachment sites available. It is likely that specific DNA sequences are preferred by the eliciting compounds. The sequences previously utilized in crystallographic modeling are authentic binding complexes [18,19] and are capable of in vivo DNA complexing especially in regions near origin of gene transcription sites such as the promoter site (above) for the *Pisum sativum* gene that codes for HMG A. HMG A codes for a transcription factor that becomes suppressed in the Pea-*Fusarium* interaction [3]. The nonhost plant response is a strong and reliable plant disease resistance defense against the challenges from an array of microbes and particularly against plant pathogenic organisms that have not evolved as pathogens of the particular plant species in question [23].

The “nonhost” disease resistance response of plants generally comprises multiple defense genes (PR genes) [24,25,26]. The identification of PR genes does not lend itself to conventional genetic analysis as was possible with the “R genes” of race-specific resistance reactions.

Race-specific resistance responses of the “Gene-for gene” interactions in pea lines can successfully match up the specific plant R genes with the pathogen’s corresponding “Avr gene.” The resulting response is multigenic and the ultimate activated genes apparently over-lap with some of the “nonhost” response (PR) genes in developing race-specific resistance [26,27,28]. The multiple-gene nonhost-resistance response expectedly employs multiple elicitors in activating multiple PR genes, as PR genes have been mapped to multiple plant host chromosomes [27].

These elicitors originate from multiple biological sources as diverse as pollen grains and animal cells. Two “biological” signals released from fungi have been investigated, namely chitosan and DNase [7,29,30], in the model nonhost resistance system. Interactions in this system involve challenges to the pea host tissue from a fungal bean pathogen for the “nonhost” response [7] and from a true fungal pea pathogen for the “susceptible” response [31].

**Figure 2:**
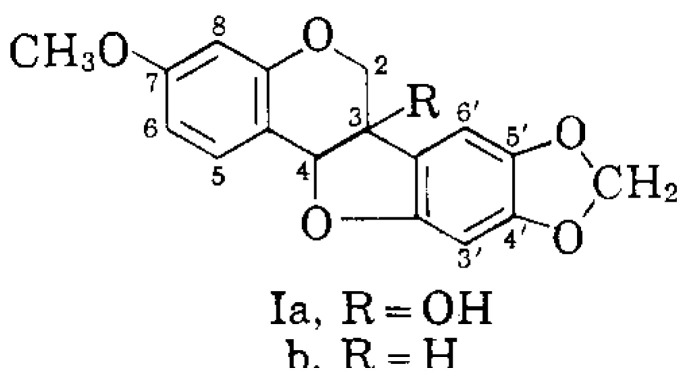
The structural formula of pisatin [33]. Structural formula derived from Perrin, D. R. and Bottomley W., Studies on phytoalexins. V. The structure of pisatin from *Pisum sativum* L. Australian J. Biol. Sci. 1962 *14*: 336–340 [33].

The biochemical functions of major PR plant genes are mostly known [32] and various mechanisms for the gene-inductions in peas have been reported [7]. The induced RNA also codes for additional antifungal proteins peptides called “defensins” found to be strongly anti-fungal [11,12]. Smaller compounds called phytoalexins (e.g., pisatin) (Figure 2) [33] are newly synthesized in plants following pathogen challenge. The pisatin pathway is activated by an array of DNA-specific agents [6,8] and by many non-pathogenic organisms.

Virulent organisms contacting plant tissue trigger some of the same responses as the avirulent pathogens [3]. The development of “complete nonhost resistance” in response to the avirulent pathogen is distinguished by a more rapid response and thus a rapid threshold of PR gene expression. [7,31]. The early defense response of plant cells is also influenced by a release of fungal DNase [7,9,29], which serves both as a signal for non-host resistance and as a known factor in suppressing fungal growth. PR gene products cause a slowing of fungal growth, as DNase accumulates in the fungal cells. The single DNA strand nicking action may terminate fungal growth by disabling cell division [29]. Both chitosan and DNase [9] function after entering the plant cell and subsequently into the plant nucleus.

### 1.4. Production of PR Gene Proteins and Pisatin

Alterations of DNA [7], including intercalation of base pair substitutions, DNA repair, DNA cleavage [6], and DNA crosslinking [34], can influence transcription [35]. The mechanism of cellular action is known for a number of the chemical compounds that were investigated and identified in searches for DNA compounds that kill cancer cells. In pea tissue, the search was for compounds that activate defense responses that occur prior to detectable cell death.

### 1.5. Natural Components

Although the chitosan macro-molecule is a long polymer of mostly glucosamine residues, a polymeric entity of at least seven residues has shown to be optimal in inducing the production of the antifungal isoflavonoid, pisatin [36]. Chitosan heptamer is also shown by computer analysis to be of an adequate size to insert into the DNA minor groove [2]. Pisatin induction also occurs when pea endocarp tissue is challenged by these “anti-cancer” drugs. The analysis of their mechanism of action has been assisted by modeling the stereochemistry of drug/DNA complexes [37,38]. For comparison purposes, netropsin has been selected for comparing the elicitation of phytoalexin by chitosan. Other small DNA minor groove binding compounds, namely methyl blue, Hoechst 33,257, chromomycin A_3_, are variable in sequence recognizing specificities and pisatin-eliciting actions.

### 1.6. Effects of Chitosan-Oligomer and Pathogen Challenge on Animal Cells

Chitosan is also capable of stimulating the immune response of both plant defenses [7] and signaling pathways in RAW 264.7 macrophages [39]. In macrophage cultures the chitosan was proposed to “promote the production of nitric oxide (NO) and cytokinins such as tumor necrosis factors α (TNF-α) and interleukin 6 (IL-6). Chitosan also enhanced the expression of inducible nitric oxide synthase (iNOS), cyclooxygenase-2 (Cox-2) and TNF-α. Chitosan also induced the phosphorylation of extra cellular signal-regulated kinase (ERK) and other genes of the mitogen-activated protein kinases (MAKs) and phosphoinositide 3-kinases (P13K)/AKt signaling pathways that are dependent on nuclear factor (NF)-kB activation that are immune potentiators” [39].

### 1.7. Chitosan Signaling in Pea Tissue

In the non-host resistance response of pea tissue, chitosan induces resistance to a bean pathogen [7]. This response is accompanied by the activation of secondary plant pathways that allow the accumulation of the isoflavonoid, pisatin. [33,40]. The nonhost resistance response of pea may also be assisted by the presence of chitosan fragments that are themselves anti-fungal against *Fusarium solani f. sp. phaseoli* [1,36,41]. The high positive charge of chitosan in other eukaryotic cells may destabilize the natural electrical charge of fungal cell walls [42]; however, it has been established that an abundance of this polycationic molecule within fungal cells becomes inhibitory to DNA and RNA synthesis [28].

In signaling in plants, chitosan can exist as a large polymer or as oligomers of glucosamine that are seven or more residues in length. This heptamer and the larger chitosan molecule have a high affinity for DNA. A computer analysis indicated that the heptamer is capable of attaching to the DNA minor groove and its direct action on DNA alters the both the UV and CD spectra of DNA in vitro [1].

Crystal models of netropsin localized in the DNA minor groove have been reported. [16]. More is known about their DNA sequence matching beyond preferences for AT-rich areas. The review by Bhaduri [18] demonstrated the structures and DNA sequence specificities with the multiple candidate DNA groove binding compounds, compared herein as pisatin elicitors in pea tissue.

## 2. Results and Discussion

The evidence that the chitosan molecules reach the nucleus of pea or wheat tissue is based on the previous detection of radio-labeled chitosan in nuclear fractions and on anti-chitosan antisera detection of chitosan in the nuclear fraction in the early hours following application [1,7]. Of the labeled chitosan entering plant cells, 19% of the label is localized within the nuclear fraction within 5 h. The nonhost resistance response of pea tissue to Fsph spores involves a combination of the signaling entities generating the complete nonhost resistance response. The accumulation of pisatin in response to the non-host pathogen (Fsph) is usually higher, but can be nearly comparable at certain concentrations 24 h following the response signaled by netropsin only (Table 1).

Netropsin-preferred DNA sequences that occur within AT-rich regions provide substantial potential for attachments. Lower concentrations of netropsin (applied with water) elicit smaller quantities of pisatin compared to Fsph spores (Table 1) with an occasional deviation spread (netropsin 0.125 mg/mL–Fsph spores treatment).

Pisatin accumulations elicited by netropsin at 0.5 mg/mL with varying Fsph spore concentrations are moderately influenced up or down depending on the Fsph spore numbers (Table 2). The positive charges of both netropsin and chitosan may cause attachment competition in living tissue. A low pisatin accumulation was observed in Table 2 when both were present in the pretreatment.

Reportedly, chitosan and netropsin both target and have in vitro credentials for inserting into the DNA minor groove [1,18]. The result of their action in vivo on pisatin production when chitosan is applied first is given in Table 3. The chitosan-lactate concentration at 0.2 mg/mL, a minimal eliciting level, was followed by applications of a range of netropsin concentrations from 1 mg/mL to 0.25 mg/mL.

The application of chitosan (0.2 mg/mL) prior to netropsin (0.5 mg/mL (Table 3) gave an increase in pisatin production, likely indicating advantageous elicitor priorities at the target sites, deviations of the elicitor in transit or other cellular factors. The chitosan-lactate product utilized herein was produced commercially to retain a maximum portion of higher molecular weight molecules, thus the challenged pea endocarp tissue is enzymatically capable of molecular cleavage resulting in an array of oligomer sizes in transit, including the heptamer which is optimal for eliciting pisatin [5,36].

### 2.1. Chromomycin

The DNA sequence preference of chromomycin differs from netropsin as more GC bases are represented in the AT-rich regions. Chromomycin has an additional ability to intercalate between DNA base pairs [16]. At concentrations of 0.5 mg/mL applied to pea endocarp tissue, chromomycin (Table 4) elicits 188 µg/g fr. wt. in comparison to 184 µg elicited by Fsph spores.

Chromomycin, individually an inducer of pisatin production, shows some additivity with Fsph spores in inducing pisatin accumulation that is more pronounced following applications of lower chromomycin concentrations (Table 5). Chromomycin elicitation in the presence of chitosan is additive at certain concentrations.

Chitosan 1 mg/mL applied following chromomycin 0.12 mg/mL treatment elevated the pisatin accumulation from 113 to 153 µg/g fr. wt. (Table 6). Chromomycin at 0.06 mg/mL applied prior to chitosan can lower the corollary chromomycin/chitosan treatment value of 81 µg to 29 µg (chromomycin/water).

Chromomycin A_3_, a groove binder DNA minor, is an effective inducer of pisatin production. Chromomycin may also be an intercalator between DNA base pairs that would add to its ability to alter DNA conformation and to induce at a lower concentration (Table 6)

Chitosan is strongly polycationic, and thus, applied alone in vivo, it also accumulates near the cell surface. Within this region, two proteins have been reported with which it attaches in vitro [14]. The relay of a transcription signal has not been resolved.

### 2.2. Other DNA Minor Groove Localizing Compounds

Pisatin induction by Hoechst 33258, a potential in vivo DNA minor groove complexing compound, is minimal and has no consistent interference with Fsph spore signaling Table 7. The cytological staining of a UV-induced fluorescent Hoechst 33258 in pea tissue verifies its potential to migrate to the nucleus. It has been shown to attach to DNA regions in vitro [18]; however, in pea tissue this attachment is apparently not suitable for eliciting pisatin production. This lack of elicitation may provide clues to the specific sequence requirements and possible environments within the plant cells.

There being multiple sites of cellular localization indicates that there are multiple targets and metabolic routes for initiating defense gene action either at the cell surface or within nuclear chromatin. The function in inducing plant defense appears to be the transcription of mRNA species that include the Pathogenesis-Related genes (PR genes) that account for the actual suppression of fungal growth.

Receptors at the cell surface or cell membrane have been reported and take multiple protein forms. Two chitosan oligomer binding proteins have been identified in tobacco and *Arabidopsis* plasma membrane by affinity chromatography. The tobacco protein is 75 kD long. The *Arabidopsis* protein is small (12 kD), suggesting that it may not be a receptor. Furthermore, unlike chitin receptors, chitosan showed no binding to CERK1 and CEBiP proteins that were involved in other signal reception routes in plants, e.g., chitin reception [14,43,44]. Moreover, Yin’s group suggested that chitosan’s cationic properties alone are not the basis of binding to the plasma membrane as binding cannot be prevented by other cationic materials such as poly-L-lysine.

Poly-L-lysine molecules and many other cationic molecules are efficient elicitors of defense responses in pea endocarp tissue, some of which are compounds that can reside in the DNA minor groove [6]. This proposed action directly on plant chromatin helps confirm that a mechanism by-passing recognition at the plant membrane may initiate a major route towards defense gene regulation. Additionally, in pea tissue, the induction of the defense response of phytoalexin production can also be mechanized by many DNA-related strategies such as groove recognition [4], intercalation [6], strand cross-linking [34] or disruption of nuclear proteins [3,45,46]. The chitosan heptamer has an approximate size and distribution of the model compound, netropsin.

The suggested minor groove-altering mechanism for activation of the secondary pathway toward pisatin production is also likely to result from related effects on the chromatin target.

### 2.3. DNA-Fragmentation Assciated with Netropsin Treatment

**Figure 3:**
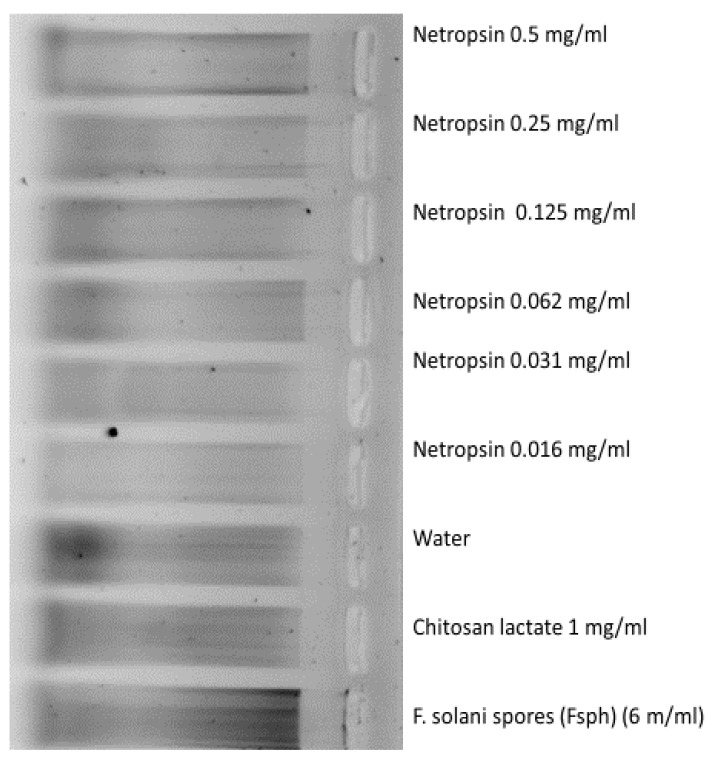
Gel separations of DNA fragments eluted from 15 µg total pea DNA in alkaline buffer and separated on agarose gel (See Methods) 2 h following the indicated treatments. Central ink dot marks the 1 kD size.

Minor size changes in DNA size distribution (See methods) occurring in pea endocarp DNA within 2-plus h following netropsin application were minimal compared with 6 × 10^6^/mL Fsph spore-treated tissue (Figure 3). These separations suggest that DNA cleavage either may not be the primary netropsin action on DNA in initiating PR gene transcription in pea endocarp tissue or the effects on DNA may be other than fragmentation.

### 2.4. The Specifics of Netropsin/DNA Dodecamer Attachment

“The netropsin amide NH furnishes hydrogen bonds in vitro to bridge DNA adenine N-3 and thymine O-2 atoms occurring in adjacent base pairs and opposite helix strands with the minor groove of the B-DNA dodecamer of the sequence C-G-C-G-A-A-T-T-C-G-C-G [16]. This binding forces open the minor groove by 0.5–2.0 A and it bends back the helix axis by 8 degrees across the region of attachment. The netropsin molecule has an intrinsic twist that favors insertion into the minor groove and experiences a small additional twist upon binding. This base specificity allows netropsin to bind preferentially with runs of four or more A-T base pairs but not G-C base pairs” [16].

The binding of netropsin to DNA causes no systematic changes in helix rotation or in base stacking—neither for unwinding nor elongating the double helix. Furthermore, it shows little to no affinity for single stranded DNA or RNA, double stranded RNA, or DNA-RNA hybrids. It does not intercalate between base pairs [16]. Chitosan oligomers in the same manner as netropsin bind DNA electrostatically. The binding of netropsin from the two cationic ends and hydrogen bonding from three amide NH groups occurs in the central part of the netropsin molecule. Its biological toxicity in animal cells results from binding to the B-DNA double helix and interfering with both replication and transcription [44].

Similarly, chitosan can suppress fungal growth by interfering with DNA and RNA synthesis [2]. The DNA affinity of netropsin is primarily due to DNA at AT-rich segments causing widening of the minor groove [37,38], indicating that netropsin differs in action from repressors and control proteins that target the DNA major groove. The binding of chitosan to DNA slightly alters the UV monitored melting spectra of DNA in vitro [1].

The potential altering of minor groove binders to varying attachment sites suggests that an unlimited diversity of receptors probably exist within eukaryotic DNA sequences that could have multiple mechanisms for activating genes adjacent to these sites on the genomic DNA [47], possibly removing barriers to the RNA polymerase complex (RNAP) transcription of genes [48,49].

This DNA diversity relative to this minor groove target can potentially be expanded further to the variation in the presence of chromatin proteins such as histones [3] and the architectural transcription factor HMG A [46].

Transcription factors can also be influenced by associated modifications and complexing agents. This could add to the enormous diversity that must exist in receptions as the cell starts responding to the equally diverse array of incoming signals functioning in phenomenon such as the nonhost resistance response of plants. This nonhost resistance response of pea plants is signaled by many of the microbes/elicitors existing in nature.

### 2.5. Signal Reception in the Hypothesized Role of Host Chromatin

The view that the reception of a signal may occur at the chromatin (DNA) level and/or may depend on the associated base sequence can encourage research directed at refining the specificities of these match ups. Genetic engineering tools can alter both the host DNA and the proteins/factors that are part of such complexes.

The knowledge of the chemistry and optimization of these DNA/chromatin complexes has been assisted directly by cancer research that has synthesized chemical compounds with optimal cell-killing biologically effects. The use of unaltered netropsin was proposed to suppress or terminate cancer cells; however, some drugs, such as netropsin, proved to be too toxic for clinical use. In contrast, the DNA minor groove targeting in plants provides a tool for following a specific system for understanding how such compounds can activate the plant secondary metabolism pathways that develop disease resistance. Newer modifications of netropsin [37] can selectively recognize mixed A·T and G·C bp sequences of DNA and can have different ways to recognize mixed sequences of DNA by modifying AT-selective heterocyclic cations.

Chemical modifications of natural compounds with the potential to insert into the DNA minor groove would expectantly be able to change their specificity along the sequences of the DNA minor groove and consequently change the optimization of the defense gene respond. In nature, chitosan exists primarily as large molecules but can be subject to enzymatic degradation to develop the optimum heptamer-plus size that is hypothesized to localize in the plant host’s DNA minor groove in addition to binding to other DNA regions.

## 3. Experimental Section

### 3.1. Elicitor Compound Sources

Netropsin dihydrochloride, Santa Cruz Biotechnology Inc., Dallas, TX, USA; Hoechst 33257, chromomycin A_3_, Sigma Chemical, St Louis. MO, USA; chitosan lactate Vanson/Halo, Redmond, WA, USA.

### 3.2. Plant and Pathogen

Immature pea pods (2 cm) were harvested from *Pisum sativum* cv. Samish plants grown in sand in the greenhouse (12 h Light) as described [29]; briefly, all fungi were cultured on potato dextrose agar (PDA) (Difco) supplemented with pea pods (5 g/L). Spores were harvested from 3-week-old cultures and suspended in sterile water. Fungal cultures in this study were *Fusarium solani f. sp. phaseoli* (ATCC no.38135) (Fsph) and *F. solani f. sp. pisi* (ATCC no. 38136) (Fspi) from bean and pea, respectively.

### 3.3. Elicitor Treatments and Pisatin Quantization

Immature pea pods (2 cm) were separated into halves with a smooth spatula. Treatments (20 µL/pod half) were applied and distributed to the exposed endocarp surface. Following 24 h at 100% humidity and 20 °C, the pod halves were submerged into 5 mL of hexanes for 24 h. The hexanes were volatized away and the residual material containing pisatin was dissolved in 95% ethanol and quantitated at UV 309 nm in a spectrophotometer as described [10].

Test elicitors were applied to the endocarp surface of immature greenhouse-grown pea pods. Elicitor treatments to the pea tissue endocarp allowed the initial treatment 30 min uptake period prior to the second application. DNA damage was assessed at 2 h [10]. Comparison treatments included elicitation by high molecular weight chitosan-lactate (Vanson/HaloSource Inc., Redmond, WA, USA) spore suspensions of the bean pathogen, *Fusarium solani f. sp. phaseoli* (Fsph) and de-ionized water.

### 3.4. DNA Damage Assessment

DNA damage/size changes were assessed following nucleic acid extraction. Fifteen µg of DNA was encumbered in an alkaline buffered (30 mM NaOH; 8 mM EDTA) chromatin grade 1% molten agarose, the solidified disc was overlaid with alkaline buffer for 72 h, and the overlay-recovered DNA was separated on 1% agarose gels for electrophoresis.

## 4. Conclusions

Currently, there is much interest in events occurring in the cytoplasm that may send signals via cascades to the nucleus, which may affect cellular transcription factors that control the activation of PR genes. Such action must be sufficient to activate as many as 20 PR genes [31,32] present on various plant chromosomes [27]. The many receptor(s) for this action must be pre-existing in plant cells with specificity for a large number of effectors. That is, an explanation is needed for the enormous variety of effectors/elicitors originating from the multiplicity of pathogens capable of inducing the nonhost resistance response of plants. Alternately, an explanation may reside within the DNA of plant chromatin with an unlimited diversity of base sequences, minor groove/major shapes, and multiple combinations of nuclear proteins [6]. Plant chromatin may be capable of many beneficial structural revisions that can activate polymerase transcriptions of stalled or silent PR genes. Furthermore, such features would also be generally necessary to rapidly affect the diversity of multiple chromosomal sites containing the PR genes [7,31]. Useful insights may come from the advanced knowledge related herein on how the DNA minor groove can be influenced by using the netropsin, chitosan, and other molecules with groove binding potential. Since DNA minor groove targeting can cause shape changes in specific attachment sites along the DNA minor groove, it is suggested that they could account for part of the changes occurring in nature that generate the nonhost resistance response of peas.

## Figures and Tables

**Table 1:** The interaction of Netropsin with spores of *Fusarium solani f. sp. phaseoli* (Fsph spores) on the production of the phytolexin, pisatin. Pea endocarp tissue was pretreated with 20 µL water or netropsin concentrations were followed after 30 min by 20 µL of Fsph spores (4 × 10^6^/mL) or water. Treatments were replicated twice. Pisatin was extracted from endocarp tissue after 24 h with hexane and quantitated at 309 nm (see Methods). + indicates the observed intensity.

Pre-Treatment	Secondary Trmt in 30 min	Pisatin µg/g fr. wt. 22 h	Hypersensitive Response at 24 h
Netropsin 0.5 mg/mL	Fsph spores	40.8 ± 5.0	+++
Netropsin 0.5 mg/mL	water	26.3 ± 1.6	+++
Netropsin 0.25 mg/mL	Fsph spores	22.6 ± 2.5	+++
Netropsin 0.25 mg/mL	water	2.1 ± 0.1	+++
Netropsin 0.125 mg/mL	Fsph spores	36.1 ± 24.0	++
Netropsin 0.125 mg/mL	water	2.7 ± 1.6	++
Water	Fsph spores	34.7± 8.2	++

**Table 2:** Induction of pisatin production employing concentrations of netropsin and chitosan previously signaling marginal levels of pisatin and 18 C incubation temperatures following pea tissue treatments were employed to reveal possible symbiotic actions. Treatments were applied to pea endocarp tissue at 20 µL/pod half. Pisatin extracted in hexane was quantitated at 309 nm. Fsph = *Fusarium solani f. sp. phaseoli* spores. Hypersensitivity was arbitrarily represented with the + symbol to indicate the relative intensities.

Pre-Treatment 20 µL/Pod Half	Secondary Trmt in 30 min 20 µL/Pod Half	Pisatin µg/g fr. wt. 24 h	Hyper. Response 24 h
Netropsin 0.5 mg/mL	Fsph spores (3 × 10^6^/mL)	56.7 ± 3.8	++
Water	Fsph spores (3 × 10^6^/mL)	101.3 ± 22.7	++
Netropsin 0.5 mg/mL	Fsph spores (7.5 × 10^5^/mL)	39.0 ± 3.9	+++
Water	Fsph spores (7.5 × 10^5^/mL)	57.0 ± 15.8	+++
Netropsin 0.5 mg/mL	Fsph spores (3.7 × 10^5^/mL)	43.8 ± 2.6	+
Water	Fsph spores (3.7 × 10^5^/mL)	35.3 ± 6.1	++
Netropsin 0.5 mg/mL	Fsph spores (1.8 × 10^5^/mL)	24.2 ± 5.0	++
Water	Fsph spores (1.8 × 10^5^/mL)	52.6 ± 10.1	++
Netropsin 0.5 mg/mL			
Fsph spores 3 × 10^6^/mL)	--------------------------------	48.9 ± 7.0	+++
Water	--------------------------------	---------------	+
Netropsin 0.5 mg/mL			
Chitosan 0.5 mg/mL	-------------------------------	10.7 ± 4.9	++

**Table 3:** Pisatin production by netropsin concentrations following pretreatment with low chitosan treatments, 20 µL volumes of all treatments of pea endocarp tissue per pod half. Hypersensitive response was estimated arbitrarily by +. Pisatin production was quantitated by UV spectrophotometer at 309 nm.

	Secondary Trmt in 30 min	Pisatin µg/g fr. wt. 24 h	Hypersensitivity Response 24 h
Chitosan 0.2 mg/mL	Netropsin 1 mg/mL	50.2 ± 3.2	+++
Water	Netropsin 1 mg/mL	22.5 ± 0.9	+++
Chitosan 0.2 mg/mL	Netropsin 0.5 mg/mL	38.7 ± 0.9	+++
Water	Netropsin 0.5 mg/mL	13.7 ± 0.1	++
Chitosan 0.2 mg/mL	Netropsin 0.25 mg/mL	27.2 ± 3.4	++
Water	Netropsin 0.25 mg/mL	14.2 ± 5.2	+++
Chitosan 0.2 mg/mL	Netropsin 0.12 mg/mL	9.8 ± 1.3	++
Water	Netropsin 0.12 mg/mL	----------	+
Fsph spores (2.4 × 10^6^)	----------------------------	244.4 ± 27.4	+++
Water	----------------------------	----------------	Light
Chitosan 0.2 mg/mL	----------------------------	0.9 ± 9.27	++

**Table 4:** Effect of chromomycin on pisatin production. All treatment applications volumes were 20 µL/pod half. Pisatin accumulations at 24 h were quantitated at UV 309 nm. + indicates the observed intensity.

Treatment (20 µL Volume)	Pisatin µg/g fr. wt.	Hypersensitivity
Chromomycin 2.0 mg/mL	69.9 ± 1.0	+++
Chromomycin 1.0 mg/mL	133.5 ± 11.2	++
Chromomycin 0.5 mg/mL	188.3 ± 3.5	+
Chromomycin 0.25 mg/mL	70.9 ± 33.5	+++
Chromomycin 0.125 mg/mL	106.1 ± 16.1	++
Chromomycin 0.06 mg/mL	77.3 ± 7.9	+++
Chromomycin 0.03 mg/mL	10.9 ± 10.9	+
Chromomycin 0.015 mg/mL	8.5 ± 7.1	+
Water	----------------	+
Fsph spores (4.5 × 10^6^)	184.7 ± 42.7	++
Chitosan 1 mg/mL	8.6 ± 4.6	+++
Netropsin 0.5 mg/mL	17.8 ± 1.9	+++

**Table 5:** Chromomycin concentrations were compared with and without a constant concentration of Fsph spores as elicitors of pisatin. All treatments were in volumes of 20 µL/pod half.

Pre-Treatment 20 µL/Pod Half	Secondary Trmt in 30 min 20 µL/Pod Half	Pisatin µg/g fr. wt. 24 h
Chromomycin 0.5 mg/mL	Fsph spores (6.4 × 10^6^/mL)	271.1 ± 23.3
Chromomycin 0.5 mg/mL	Water	111.2 ± 9.2
Chromomycin 0.25 mg/mL	Fsph spores (6.4 × 10^6^/mL)	248.8 ± 10.8
Chromomycin 0.25 mg/mL	Water	109.8 ± 3.8
Chromomycin 0.12 mg/mL	Fsph spores (6.4 × 10^6^/mL)	291.0 ± 9.5
Chromomycin 0.12 mg/mL	Water	58.9 ± 9.9
Chromomycin 0.06 mg/mL	Fsph spores (6.4 × 10^6^/mL)	270.9 ± 17.5
Chromomycin 0.06 mg/mL	Water	53.7 ± 6.3
Chromomycin 0.03 mg/mL	Fsph spores (6.4 × 10^6^/mL)	259.7 ± 28.4
Chromomycin 0.03 mg/mL	Water	78.3 ± 43.2
Water	Fsph spores (6.4 × 10^6^/mL)	210.6 ± 34.9
Water	Water	----------------

**Table 6:** The combined action of chromomycin and chitosan in elicitation of pisatin production. Pea endocarp halves received 20 µL volume of all treatments as indicated and pisatin was quantified as above.

Pre-Treatment 20 µL/Pod Half	Secondary Trmt in 30 min 20 µL/Pod Half	Pisatin µg/g fr. wt.
Chromomycin 0.12 mg/mL	Chitosan 1 mg/mL	153.0 ± 87.1
Chromomycin 0.12 mg/mL	Water	113.2 ± 22.1
Chromomycin 0.06 mg/mL	Chitosan 1 mg/mL	81.9 ± 1.2
Chromomycin 0.06 mg/mL	Water	29.9 ± 8.9
Chromomycin 0.03 mg/mL	Chitosan 1 mg/mL	33.4 ± 3.5
Chromomycin 0.03 mg/mL	Water	29.7 ± 20.6
Chromomycin 0.015 mg/mL	Chitosan 1 mg/mL	20.6 ± 3.0
Chromomycin 0.015 mg/mL	Water	2.1 ± 0.0
Chromomycin 0.007 mg/mL	Chitosan 1 mg/mL	43.2 ± 22.9
Chromomycin 0.007 mg/mL	Water	2.4 ± 0.5
Water	Chitosan 1 mg/mL	9.4 ± 4.5
Water	Water	0.0 ± 0.0

**Table 7:** The effect of Hoescht 33258 and/or Fsph spores (3 × 10^6^) on the production of pisatin in pea tissue after 24 h.

Pre-Treatment	Secondary Treatment 30 min	Pisatin µg/g fr. wt. 24 h
Hoechst 33258 1 mg/mL	Fsph spores	102 ± 7
Hoechst 33258 1 mg/mL	Water	9 ± 1
Hoechst 33258 0.5 mg/mL	Fsph spores	91 ± 7
Hoechst 33258 0.5 mg/mL	Water	3 ± 2
Hoechst 33258 0.25 mg/mL	Fsph spores	116 ± 26
Hoechst 33258 0.25 mg/mL	Water	1 ± 0.6
Hoechst 33258 0.125 mg/mL	Fsph spores	90 ± 5
Hoechst 33258 0.125 mg/mL	Water	2 ± 0.9
Water	Fsph spores	84 ± 21
Water	Water	---------
Chitosan lactate 1 mg/mL	Fsph spores	125 ± 62
Chitosan lactate	Water	9 ± 2
